# Determination of Binary Gas Mixtures by Measuring the Resonance Frequency in a Piezoelectric Tube

**DOI:** 10.3390/s22041691

**Published:** 2022-02-21

**Authors:** Kanchalar Keeratirawee, Peter C. Hauser

**Affiliations:** 1 Department of General Sciences, King Mongkut’s Institute of Technology Ladkrabang Prince of Chumphon Campus, Chumphon 86160, Thailand; k.keeratirawee@unibas.ch; 2 Department of Chemistry, University of Basel, Klingelbergstrasse 80, 4056 Basel, Switzerland

**Keywords:** speed of sound, resonance frequency shift, N_2_, O_2_, CO_2_, He

## Abstract

The composition of gas mixtures may be determined via changes of the speed of sound. As this affects the resonance frequency of the gas inside a tube, indirect measurements through a frequency analysis are also possible. It is demonstrated that this may be carried out with unprecedented simplicity by the novel employment of a piezoelectric tube which serves at the same time as a resonance tube and as transducer into the electronic domain. Experiments were run using a simple diecast aluminum box as the measuring cell, inside which the piezoelectric tube made from lead zirconium titanate with 30-mm length and 5.35-mm inner diameter was suspended. A small loudspeaker placed into the cell served for excitation of the resonance. Peak frequencies between 3910 and 14,590 Hz (for pure CO_2_ and He, respectively) were obtained. Two component mixtures of O_2_/N_2_, CO_2_/N_2_, and He/N_2_ at various composition were tested. A linear frequency change from 4790 to 5100 Hz was observed when going from pure O_2_ to pure N_2_.

## 1. Introduction

The speed of sound, *c*, in a pure gas is dependent on its molecular mass (*M*) according to the following equation:

(1) $$c=\sqrt{\frac{\gamma RT}{M}}$$

*γ* is the adiabatic index (which depends on the nature of the gas), *R* is the universal gas constant, *T* is the absolute temperature. For mixtures of different gases, the speed of sound is determined by the mean molecular mass, *M_mix_* [1].

(2) $$c_{mix}=\sqrt{\frac{\gamma_{mix}RT}{M_{mix}}}$$

For biatomic gases *γ* is nearly constant at a value of 1.40 [2], but for mixtures involving other types of gases *γ* also shows a dependence on the composition [1].

While not widely used, it is therefore possible to determine the composition of gas mixtures from the measurement of the speed of sound. One possibility is to use the time of flight method, in which the transit time of a pulse is measured (see for example [3,4]). An alternative method is the resonance frequency shift method. The principle was exploited as early as 1913 in a device designed by Fritz Haber (who is better known for the development of the artificial nitrogen fixation process) for the detection of methane build-up in underground mining [5]. This worked without electricity, the miners would simply listen to the sound from the whistle. As the frequency of the sound coming from a whistle is dependent on the composition of the gas a change in pitch would alert them to the danger. More recently electronic devices based on this principle have been reported. Garret [1] reported an analyzer for hydrogen and methane based on a copper cylinder as the resonator, which was fitted with a loudspeaker and microphone for creating and pick-up of the sound waves. Ruffine and Trusler [6] described a similar arrangement designed for use in gas pipelines employing two planar piezoelectric transducers at the ends of a cylindrical resonator for sound creation and pick-up. Suchenek and Borowski [7] employed a slightly different approach for sensing of Ar and CO_2_ in nitrogen in that the acoustic wave was produced by the photoacoustic effect with the help of carbon black. However, instead of the intensity measurement, as employed in photoacoustic spectrometry, the change in the resonance frequency was monitored. Cicek et al. [8] described a system for CO_2_ and CH_4_ measurements based on a ring resonator fitted with phononic crystals, which employed an ultrasonic transducer for sound generation and a microphone for pick-up. A commercial instrument (BGA244) for determining the ratio of binary gas mixtures based on the change of the resonance frequency in a cylindrical cavity is available from Stanford Research Systems (www.thinksrs.com, accessed 20 January 2022).

For a gas contained in a tube, the fundamental resonance frequency, *f*, along its length, *L*, is given by the following equation:

(3) $$f=\frac{c}{2L}$$

For a tube which is open at both ends, an end correction factor has to be applied [9,10]:

(4) $$f=\frac{c}{2\left(L+0.6r\right)}$$

where *r* is the radius of the tube and the factor 0.6 is an approximation. 

To implement the measurement two transducers are normally required. A frequency scan is carried out with some kind of a loudspeaker or ultrasonic transducer to excite the resonance and a microphone to determine the peak in the spectrum. In photoacoustic spectroscopy similar resonance tubes fitted with a microphone are also employed, but the acoustic signal is produced by the absorption of light energy by the gas. The modulation of the light intensity at the resonance frequency of the measuring cell provides an inherent amplification of the signal before pick up by the microphone as transducer. Recently Keeratirawee and Hauser [11] demonstrated that it is possible to implement gas phase photoacoustics with a resonance tube made from piezoelectric material (lead zirconium titanate, PZT) as the resonance body. The tube was coated with silver electrodes on the in- and outside and could serve directly as transducer from the acoustic to the electric domain, thus eliminating the need for coupling a microphone to the measuring cell.

In this study, the determination of the composition of binary gas mixtures by evaluating their resonance frequency in such a piezoelectric tube is reported. To our knowledge, the use of such a device, to serve at the same time as the resonance body and the transducer of the signal into the electrical domain, has not previously been reported.

## 2. Materials and Methods

### 2.1. Materials

The piezoelectric tube (PT-130.10), with 30-mm length and 6.35-mm outer and 5.35-mm inner diameter, was obtained from PI Ceramic (Lederhose, Germany). The signal from the piezoelectric transducer was amplified with an operational amplifier (OPA602AP, Texas Instruments, Dallas, TX, USA) fitted with a feedback resistor of 6.8 kΩ. The small loudspeaker (diameter = 15 mm)(KSSG1508) was purchased from Farnell (Zug, Switzerland). A purposed made audio amplifier circuitry (LM386N, Texas Instruments) was employed as a loudspeaker driver. The sine wave was produced by the function generator built-in into the lock-in amplifier employed for acquiring the frequency spectra (MFLI from Zurich Instruments, Zurich, Switzerland). The diecast aluminum case (92 × 38 × 31 mm) used as measuring cell was a product of Hammond Manufacturing (Eddystone 27969PSLA, Guelph, Canada). Swagelok (Wohlen, Switzerland) fittings and tubes were employed to allow passing of the test gases (N_2_, O_2_, CO_2_, and He, obtained from PanGas, Pratteln, Switzerland) through the cell. Binary gas mixtures were created by setting appropriate flow rates from two tanks with mass flow controllers (models F-201CV-200-AAD-22-V and Fe201CV-1K0-AAD-22-V with maximum flow rates of 200 and 1000 mL/min, purchased from Bronkhorst, Aesch, Switzerland). Silver loaded epoxy was obtained from RS Components (Wädenswil, Switzerland).

### 2.2. Measurement Set-Up

**Figure 1:**
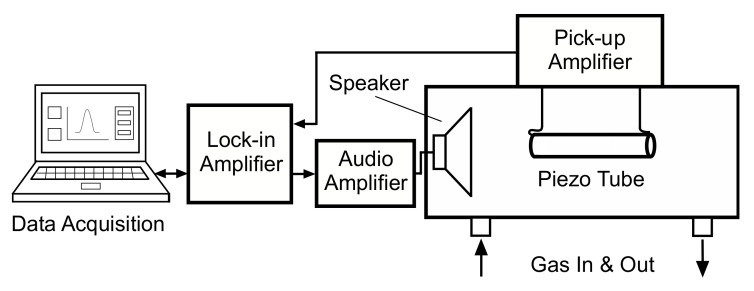
Schematic drawing of the piezo transducer set-up for evaluating the gases (not to scale).

**Figure 2:**
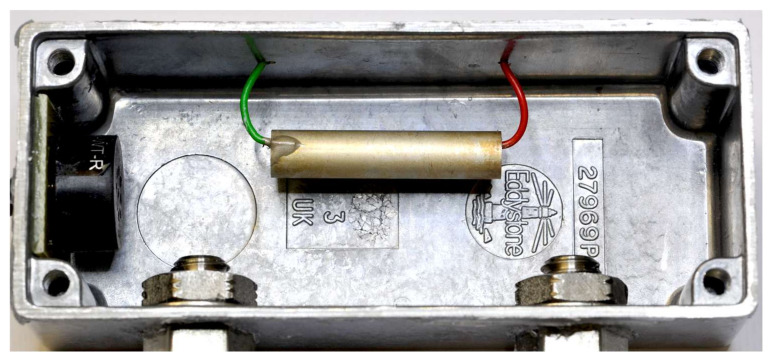
Photograph of the measuring cell with the suspended piezoelectric transducer in the center, the loudspeaker on the left and the gas in- and outlet at the bottom.

The set-up is shown schematically in Figure 1 and a photograph of the cell in Figure 2. The piezoelectric transducer tube was placed inside a small hermetically sealed case serving as the measuring cell. This also contained the loudspeaker for excitation and in- and outlet for the test gases. The piezoelectric tube was freely suspended by the two wires making the electrical connections. These were attached to the inside and outside electrodes of the tube using a small amount of silver loaded epoxy. The piezoelectric output signal was amplified with an operational amplifier. The transimpedance configuration, which was found to be superior to the voltage amplifier configuration in our previous study [11], was again employed. A computer based lock-in amplifier and computer controlled mass-flow controllers employed for producing the test gas mixtures complete the system.

## 3. Results and Discussion

### 3.1. Resonance Frequency

**Figure 3:**
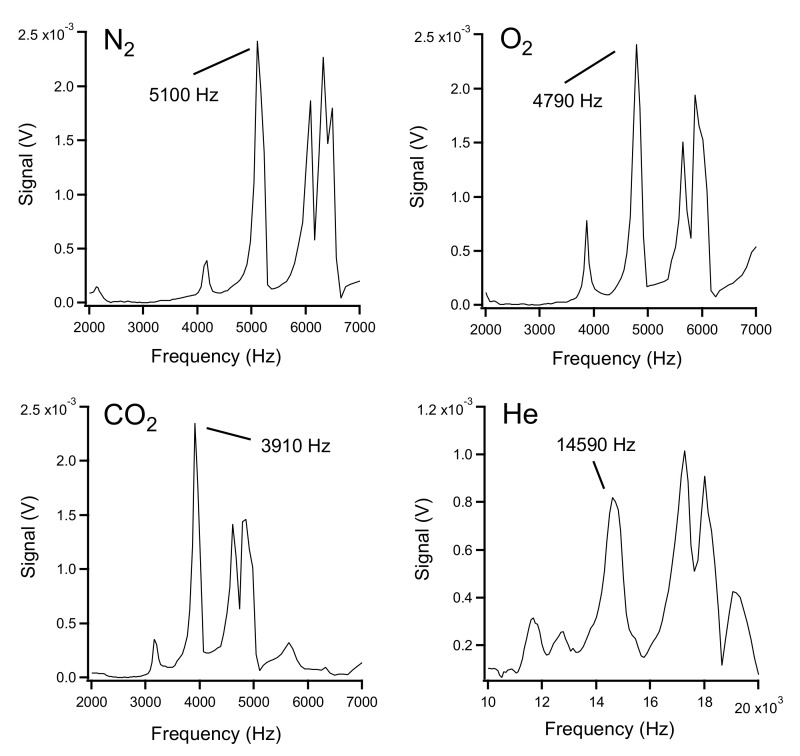
Resonance spectra for the four pure gases.

Frequency spectra obtained with the set-up for pure nitrogen, oxygen, carbon dioxide, and helium are shown in Figure 3.

As can be seen, a number of peaks were obtained with similar patterns for the four gases, but they were shifted in frequency. The multitude of peaks must be due to resonances in the cell other than the longitudinal wave inside the tube itself. The speed of sound in pure nitrogen (N_2_), oxygen (O_2_), carbon dioxide (CO_2_), and helium (He) gas at 0 °C is 334, 316, 259, and 965 m/s, respectively [12]. According to Equation (4), fundamental resonance frequencies of 5284, 4999, 4097, and 15266 Hz, respectively, result from these values. Corresponding peaks can indeed be found in the spectra for the four gases at approximately 5100, 4790, 3910, and 14590 Hz, respectively. For nitrogen, oxygen, and carbon dioxide these are the most prominent peaks, while for helium the intensity is slightly lower than that of other peaks observed. Note, that the experimentally determined frequencies are somewhat lower than the theoretical values. This must be due to the fact that the correction factor of 0.6 in Equation (4) is only an approximation. Furthermore, the experiments were carried out at room temperature, and therefore the temperature did not correspond to the 0 °C for the above quoted literature values of the speed of sound.

### 3.2. Measurements of Mixtures

**Figure 4:**
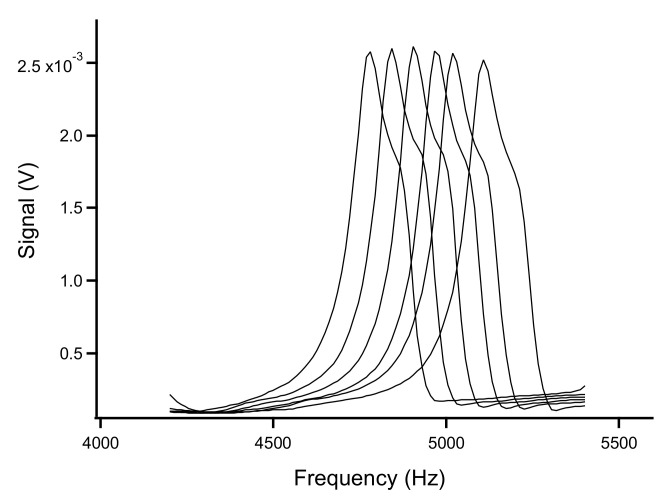
The resonance peak for O_2_/N_2_ mixtures. From left to right: 100%, 80%, 60%, 40%, 20%, and 0% oxygen.

In order to evaluate the suitability of the set-up for the determination of the composition of gas mixtures, frequency spectra for different fractions of O_2_, CO_2_, and He in N_2_ were acquired. The effect of the composition on the fundamental resonance peak is shown for O_2_/N_2_ mixtures between 100% oxygen and 100% nitrogen in Figure 4. A frequency change approximately from 4790 to 5100 Hz was observed when going from pure O_2_ to pure N_2_.

**Figure 5:**
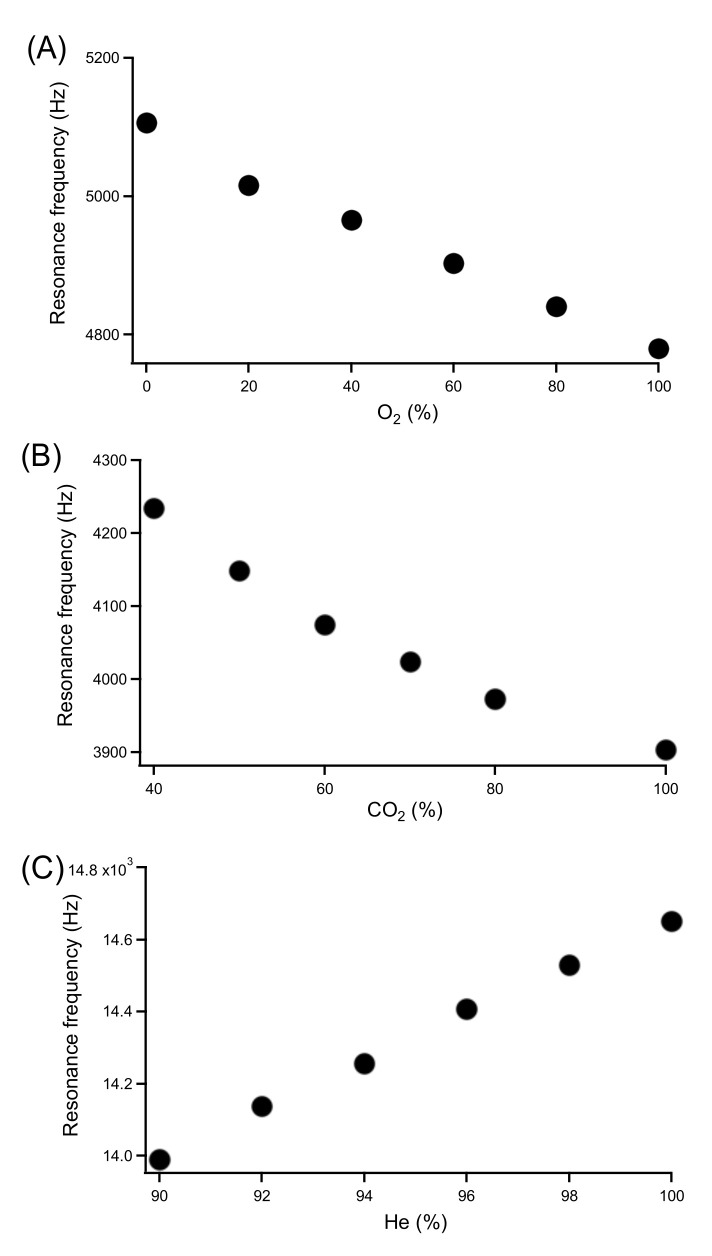
The relationship between resonance frequency and gas composition. (**A**) O_2_, (**B**) CO_2_, and (**C**) He in N_2_.

Plots of the determined peak frequencies for mixtures of O_2_, CO_2_, and He with nitrogen are shown in Figure 5. For the mixtures of O_2_ and CO_2_ in N_2_, the trend to lower frequencies with higher fractions of the gas added to nitrogen expected from Equation (2) due to their higher molecular masses was confirmed. For He, the trend is the opposite due to the low atomic mass of the noble gas. The plot for the O_2_/N_2_ mixtures (Figure 5A) is close to linear, as is expected from Equation (2) when only diatomic gases are involved. In contrast, for CO_2_/N_2_ mixtures (Figure 5B) a pronounced nonlinearity was observed. This also corresponds to the expected as the adiapatic index, *γ*, in Equation (2) is not a constant for these mixtures. Such a non-linearity is also expected for the He/N_2_ mixtures, but presumably due to the limited range explored they are not apparent in Figure 5C.

## 4. Conclusions

The preliminary results shown in this communication demonstrate the potential of the novel approach of using a piezoelectric tube for the determination of the composition of gas mixtures. Combining the two functions of the resonance tube and the transducer results in a set-up of unprecedented simplicity. More work is required to establish the scope and sensitivity of the method. Given the temperature dependence of the speed of sound (see Equation (2)), for precise measurements it will also be necessary to control the temperature or to measure it and apply a correction to the measured frequency.
